# Non-Invasive Imaging Through Scattering Medium by Using a Reverse Response Wavefront Shaping Technique

**DOI:** 10.1038/s41598-019-48788-9

**Published:** 2019-08-22

**Authors:** Abhijit Sanjeev, Yuval Kapellner, Nadav Shabairou, Eran Gur, Moshe Sinvani, Zeev Zalevsky

**Affiliations:** 1 Faculty of Engineering and the Institute for Nanotechnology and Advanced Materials, Bar-Ilan University, Ramat-Gan, 5290002 Israel; 2 EKB Technologies Ltd., Bat Yam, 5951301 Israel; 3 Azrieli College of Engineering, Jerusalem, 9103501 Israel

**Keywords:** Adaptive optics, Optical imaging

## Abstract

Fundamental challenge of imaging through a scattering media has been resolved by various approaches in the past two decades. Optical wavefront shaping technique is one such method in which one shapes the wavefront of light entering a scattering media using a wavefront shaper such that it cancels the scattering effect. It has been the most effective technique in focusing light inside a scattering media. Unfortunately, most of these techniques require direct access to the scattering medium or need to know the scattering properties of the medium beforehand. Through the novel scheme presented on this paper, both the illumination module and the detection are on the same side of the inspected object and the imaging process is a real time fast converging operation. We model the scattering medium being a biological tissue as a matrix having mathematical properties matched to the physical and biological aspects of the sample. In our adaptive optics scheme, we aim to estimate the scattering function and thus to encode the intensity of the illuminating laser light source using DMD (Digital Micromirror Device) with an inverse scattering function of the scattering medium, such that after passing its scattering function a focused beam is obtained. We optimize the pattern to be displayed on the DMD using Particle Swarm Algorithm (PSO) which eventually help in retrieving a 1D object hidden behind the media.

## Introduction

Random scattering of light creates serious hindrance in imaging through a scattering medium like biological tissues. There was a widespread need felt to image through these scattering media for various applications ranging from biomedical to astronomical imaging. In the late 1960s, several works were suggested by Goodman *et al*.^1^, Leith and Upatnieks^2^ and Kogelnik and Pennington^3^ for imaging through random scattering medium. With the advent of technological advancements, several techniques came into existence that allowed imaging through these media, despite light scattering. Techniques such as Optical Coherence Tomography (OCT)^4^ and two photon microscopy^5,6^, use ballistic photons (photons which are not scattered) to image through the media. However, the media produces abundant scattering, and these techniques are less efficient due to lower number of ballistic photons. There are other techniques that utilize the scattered photons. Diffuse optical tomography^7^ is one such method that spatial resolution is limited, due to the scattering depth.

Speckle produced when a beam of light is incident on scattering media remains correlated even if there is a small tilt in the incident wavefront over a specific area on the media. This effect is known as ‘*Optical Memory Effect*’. It was exploited in several works to obtain this most desirous goal of imaging through the perturbed media^8–10^. Freund^11,12^ introduced this concept of using a scattering media as a lens based on speckle intensity correlation. Bertolotti *et al*.^13^ extended this idea to image a fluorescent object behind the media. Katz *et al*.^8^ introduced a single-shot imaging technique to retrieve image of a hidden object. His concept was based on the intensity correlation of the speckle being identical to that of the object. Hence, he used Fienup’s phase retrieval algorithm^14^ to reconstruct the object behind the media. However, the quality of the reconstructed image faced artifacts, mainly due to the initial guess involved in the phase retrieval algorithm. Later, Sing *et al*.^15^ came up with the idea of using a reference point source along with the object in which the point source serves as a reference. The advantage of using simple autocorrelation of the camera intensity allowed the image to be reconstructed, hence avoiding the use of a phase retrieval algorithm. Ghost imaging also helped to retrieve the information of an unknown object^16–18^. However, a reference beam was also required for its implementation.

Optical wavefront shaping technique has been proven to be a very effective technique in focusing light through a scattering medium. The discovery of Spatial Light Modulators (SLM) brought out a huge impact in this field. Optical Phase Conjugation (OPC) is one, among the wavefront shaping techniques, that is being extensively used to image through a scattering medium. Zahit *et al*.^19^ focused light through a thick chicken breast tissue using OPC, and proved that the width of the reconstructed focus spot is independent of the thickness of the sample. Holographic methods have also been used in improving images perturbed by the presence of scattering medium. One such method done by Vittorio *et al*.^20^ utilized the Brownian movement of colloidal particle to obtain multiple uncorrelated holograms, and later combined them to reduce the speckle contrast. Later through his other work^21^, he used the same technique to tackle the problem of imaging transparent samples that were hidden behind biological occluding objects in microfluidic platforms. Whereas, Melania *et al*.^22^ used the doppler frequency shift experienced by the photons scattered by the flowing colloidal particles, which do not contribute to the recorded hologram.

Vellekoop *et al*.^23^ performed wavefront shaping using an SLM to obtain a focused spot behind a scattering medium. In their optical scheme, the scattering medium is illuminated by a light source whose phase is modulated by an SLM. A camera is placed behind the scattering medium and this acts as a feedback to the SLM. Finally through optimization, a focused spot is obtained on the camera. Later, Popoff *et al*.^24^ came up with the idea of measuring the transmission matrix of a scattering media and using it to introduce the inverse phase on the SLM to get a focused spot behind the scattering media. In 2014, Youngwoon Choi^25^ demonstrated that a scattering medium can be used as a lens to image objects behind it by exploiting the transmission matrix. He performed an endoscopic imaging through a single multimode optical fiber (acts like a scattering medium) as a lens. This method requires an initial calibration to find the complete transmission matrix. There were several works done by using SLM, as a phase compensator, to image through a scattering medium^26–36^. In spite of all the advantages of all the aforementioned techniques, most of them require some sort of feedback from behind the medium, hence making it unsuitable for non-invasive imaging through the medium.

**Figure 1:**
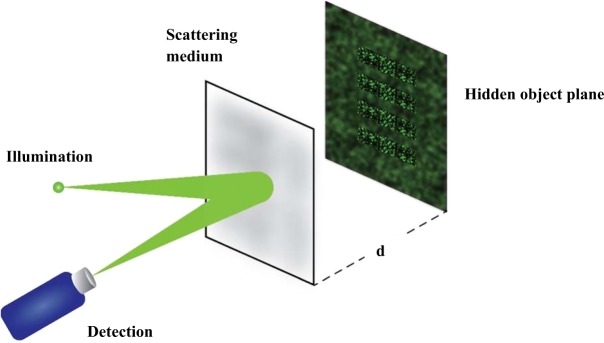
Schematic description of the proposed non-invasive technique for imaging through a scattering medium. A hidden object (barcode) placed at a ‘*d*’ distance from the media is being illuminated by a laser source. The speckle illuminating the object returns in phase opposition through the same medium. The detection is on the same side as that of the illumination making it non-invasive.

We propose a novel wavefront shaping scheme which we call ‘Reverse Response Wavefront Shaping Technique’ (RRWST), in which the illumination and detection is on the same side of the inspected tissue (*see* Fig. 1). In this paper, we introduce our modeling technique and demonstrate it by *MATLAB* simulation that we can focus light through a scattering medium in a non-invasive manner, and implement it through experiments to reconstruct a 1-D object hidden behind a scattering medium.

## Materials and Methods

### Principle

In our adaptive optics scheme, we aim to estimate the scattering function to encode the wavefront of the illuminating laser light source with an inverse scattering function of the scattering medium in such that after passing its scattering function, a focused beam is obtained. This wavefront was obtained after passing twice through the same scattering medium (on the way forward and on the way back).

In order to estimate the encoded inverse wavefront that will be focused after passing the above-mentioned scattering medium, we use the following modeling: we assume that the scattering medium can be modeled by a sequence of operators multiplications –while the first is an operator of random phase and the second is an operator of short free space propagation of distance of *dz*, being the average scattering length in the inspected tissue. This couple of operators is repeated, until the number of repeats equals to *M* = *L/dz* –where *L* is the distance of the target behind the media from the point of illumination. Thus, by modeling the 1-D case (*refer to the Supplementary material for 2-D formulation*) due to cause of simplicity, the forward scattering matrix *A* can be written as:

1 $$\begin{array}{c}A={({[{F}_{ij}]}^{\ast }[\begin{array}{cccc}{e}^{-i\pi \lambda dz{{\mu }_{j}}^{2}} & \cdots  & 0 & 0\\ \vdots  & \ddots  & 0 & \vdots \\ 0 & \cdots  & {e}^{-i\pi \lambda dz{{\mu }_{j}}^{2}} & 0\\ 0 & 0 & 0 & {e}^{-i\pi \lambda dz{{\mu }_{j}}^{2}}\end{array}][{F}_{ij}][\begin{array}{cccc}{P}_{11} & \cdots  & 0 & 0\\ \vdots  & \ddots  & 0 & \vdots \\ 0 & \cdots  & {P}_{N-1N-1} & 0\\ 0 & 0 & 0 & {P}_{NN}\end{array}])}^{M}\\ \,\,\,\,\,\,\,Free\,Space\,Propagation\,Matrix\,\,\,\,Phase\,Scattering\,Matrix\end{array}$$

where [*F*_*ij*_] and [*F*_*ij*_]^*^ are Fourier and inverse Fourier matrix. Hence,

2 $${E}_{ou{t}_{j}}({x}_{j})={A}^{t}\ast A\ast {E}_{i{n}_{j}}({x}_{j})$$

It is to be noted that the matrix *A* is unitary, since it is the product of multiplication of two unitary matrices. The operator matrix *A* was applied twice as there is a double passing through the scattering medium (on the way forward and on the way back). On the way back, the scattering is in inverse order and this is the reason that the transpose operation was applied on the matrix *A*. To find the input field distribution vector E_in_ (which e.g. can be a phase only distribution) after one passage when a focus is obtained, *i*.*e*. the operator *A* produces a delta function in the middle of the spatial axis (zero position coordinate):

3 $$[\begin{array}{c}0\\ 0\\ 1\\ 0\\ 0\end{array}]=A\ast {E}_{i{n}_{j}}({x}_{j})$$

Thus, one can extract that:

4 $${({A}^{t})}^{-1}\ast {E}_{ou{t}_{j}}({x}_{j})=A\ast {E}_{i{n}_{j}}({x}_{j})=[\begin{array}{c}0\\ 0\\ 1\\ 0\\ 0\end{array}]$$

and, therefore:

5 $${E}_{ou{t}_{j}}({x}_{j})={A}^{t}[\begin{array}{c}0\\ 0\\ 1\\ 0\\ 0\end{array}]={{A}^{t}}_{N/{2}_{J}}$$

*N* is the number of spatial sampling points along the output axis.

From Eq. 2, E_in_ equals to the middle column of the matrix operator of inverse *A*:

6 $${E}_{i{n}_{j}}({x}_{j})={A}^{-1}\ast [\begin{array}{c}0\\ 0\\ 1\\ 0\\ 0\end{array}]={{A}^{-1}}_{{\frac{N}{2}}_{j}}$$

*A* is a unitary matrix, because it is a product of two unitary matrices. *(refer to the Supplementary material for the proof)*. There is a known relation between *A* transpose (*A*^t^) and inverse of *A* (*A*^*−1*^).

7 $${A}^{-1}={A}^{\ast }$$

where * applied on a matrix *A* denoting its conjugate transpose. By combining Eqs 5, 6 and 7, we have:

8 $${E}_{ou{t}_{j}}^{\ast }({x}_{j})={E}_{i{n}_{j}}({x}_{j})$$

Hence, we obtain the desired ${E}_{i{n}_{j}}({x}_{j})$ to be applied on the SLM for having a focused spot, after 1^st^ passage through the medium. A pictorial representation of the above presented mathematical steps that can be seen as two steps described inFig. 2(a,b).

**Figure 2:**
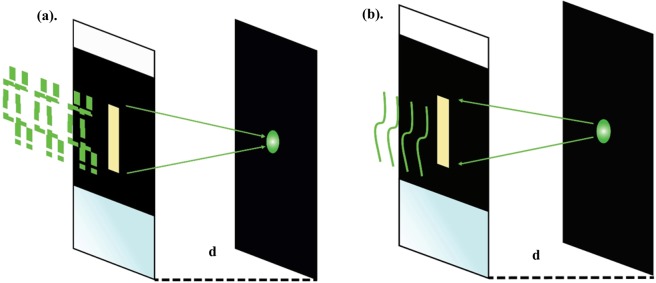
Scattering tissue is placed in between two glass slides, serving as a scattering medium. (**a**) Displays that an ideal shaped E_in_ produces a focused spot behind the media on the target plane at a distance of *d*. (**b**) In the case of (**a**), where a focus is produced, the focus spot after reflection from the target plane will pass through the medium un-scattered, but in the opposite direction. The case of (**a**) is our initial assumption. In our optimization, we measure the phase of the output light and correlate it with the input light, resulting in the estimation of that E_in_, which produces a focus spot after a single pass.

Equation 8 can be written in terms of Intensity as:

9 $${I}_{ou{t}_{j}}({x}_{j})={I}_{i{n}_{j}}({x}_{j})$$

Obviously, the above described model is an approximation and the obtained analytical result is only the starting iteration point of the distribution to be displayed on the wavefront shaping device –around which a close loop convergence process needs to be applied to achieve the focus, after the 1^st^ pass. Hence, there are two ways of obtaining the focal spot by this optimization. Either by using phase modulation –where the Eq. 8 is used as the optimizing cost function; or in an amplitude modulation scheme, by using Eq. 9. Phase modulation can be achieved by capturing the phase of the output light by using holography, and then correlating it with an input phase applied on an SLM. In an amplitude modulation (a rather easy-to-implement, experimentally), the input light with varying intensity is correlated with the intensity of the output light to obtain focus by optimization. Through *MATLAB* simulations, the validity of the method using phase modulation is verified and equally valid for amplitude modulation as well. However, to prove the concept, experimentally, amplitude modulation was chosen for more simple alignment and for ease of this experiment.

### Optimization scheme used

**Figure 3:**
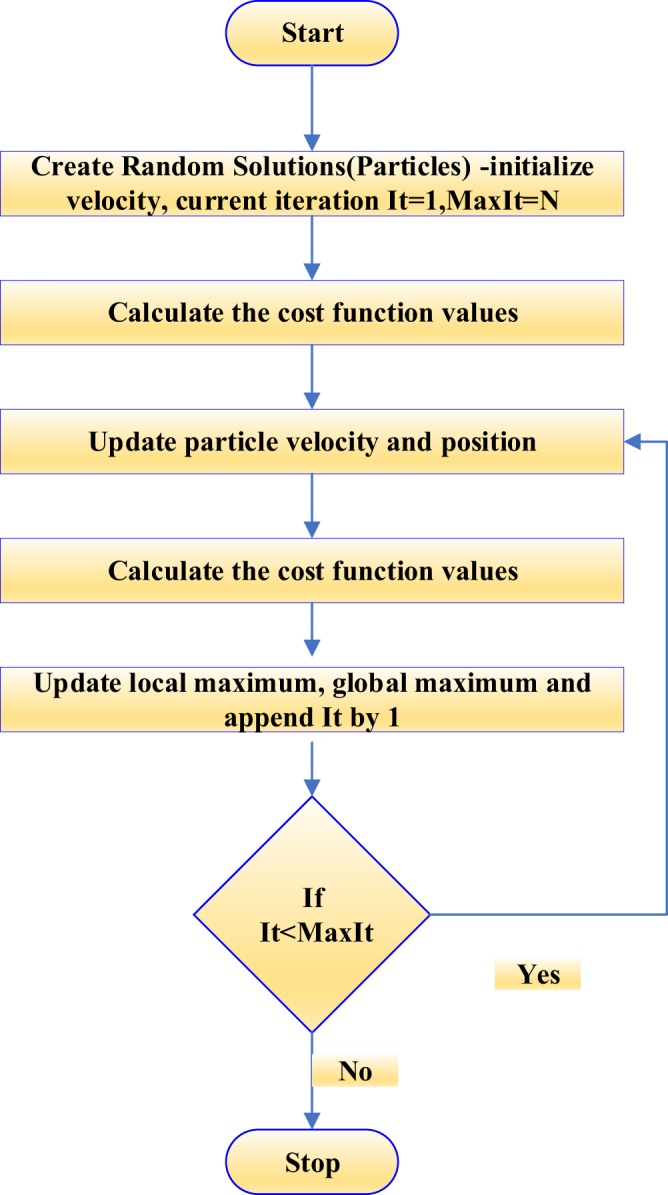
Flowchart describing the steps involved in PSO.

We used Particle Swarm Optimization Scheme (PSO) to perform the iterations. It is a stochastic optimization technique that mimics the social behavior of birds flocking together or fish schooling. It was introduced by Kennedy, Eberhart and Shi^37^. In computational sense, PSO finds an optimum solution for a problem by iteratively improving the solution from a set of candidate solutions being random guess solutions to a problem. In technical terms, these solutions are called ‘*particles*’. Each particle is assigned a *‘position’* and a *‘velocity’* vector. The search space is known as *‘swarm’*. Hence, the name PSO. The flowchart describing the steps involved in PSO is displayed in Fig. 3. The flow chart shows that the optimization is performed for a specific number of iterations called ‘*MaxIt*’. However, the termination criteria could be set by calcualting a specific cost function value, below where the iteration continues and above where the iteration stops. Our supplementary material shows simulation results based on the aforementioned criteria.

## Simulation Results

**Figure 4:**
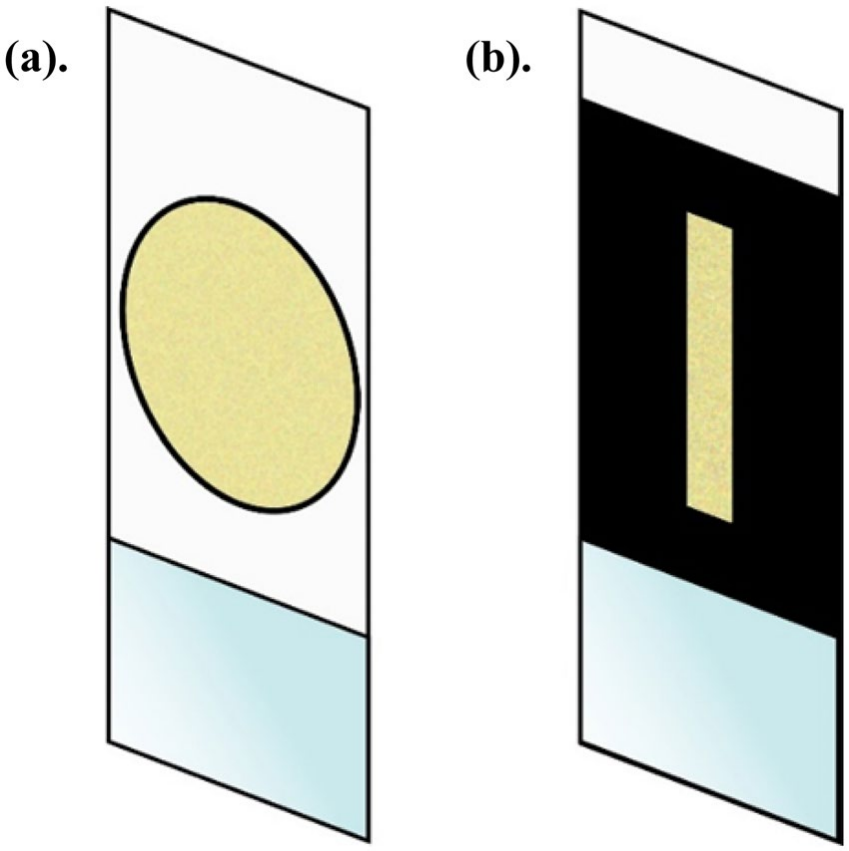
(**a**) Chicken tissue is placed between two glass slides serving as a scattering medium. (**b**) Masked version of (a) such as the effective area of illumination on the medium in the horizontal direction is very minimal –making it more like a 1-D scatterer.

*MATLAB* simulations were performed to serve as a proof of concept for our proposed (RRWST) technique. A 100 × 100 pixels for simulation was chosen. Since the modeling is in 1-D, the effective area of the illumination was kept on the scattering medium minimal, *i*.*e*. a line illumination was chosen. There are 100 pixels whose phase values can be changed from 0 to 2π along the vertical axis. Along the horizontal direction, 6-pixel columns were chosen for the line illumination. The pixel size was set at 1 µm. Ideally, it would mean that a line of light, whose phase is modulated along the vertical direction, is incident on a scattering media. Since the scattering contribution from other areas of the scattering sample is undesired, the rest of the area was blocked on the scatterer, aside from the illumination area (*see* Fig. 4). The phase modulated light (*E*_*in*_) is allowed to pass through the medium which then scatters and illuminates the target plane at 0.5 mm with speckle. The light that bounces back off of the target plane returns in phase opposition through the same medium. We simulate this situation and encode the phase of the output light (*E*_*out*_) that has passed twice through the medium. From Eq. 8, the cost function that we try to maximize is the phase correlation between *E*_*in*_ and *E*_out_^*^.

Since there are an infinite number of solutions for the optimization to converge to a spot, the solution space was resticted. In the target plane, a binary mask was introduced in the form of a barcode with alternating black and white lines. Each white space is 10 pixels in its width (5 such white spaces of 10 pixel width). This serves as the hidden object in this simulation. The aim is to focus light on this object. Each iteration would yield a focus at different spatial location. An angular spectrum propagation method was used to propagate the light from the scatterer to the object and back. A single *particle* in our scheme comprises of 100 variables, whose phase values change from 0 to 2π. It was started with 20 such random *particles*. Hence, the *population* of our *swarm* is 20. *MaxIt* is set at 4000. The algorithm starts with finding the best solution, *‘pbest’*, corresponding to each particle in the swarm. The cost function value corresponding to it is known as *‘pbsestcost’*. In the entire swarm, there would be one particle whose cost/merit function would be the best. This particle is termed as ‘*gbest*’. The cost function value corresponding to it is known as ‘*gbestcost*’. This is one iteration.

**Figure 5:**
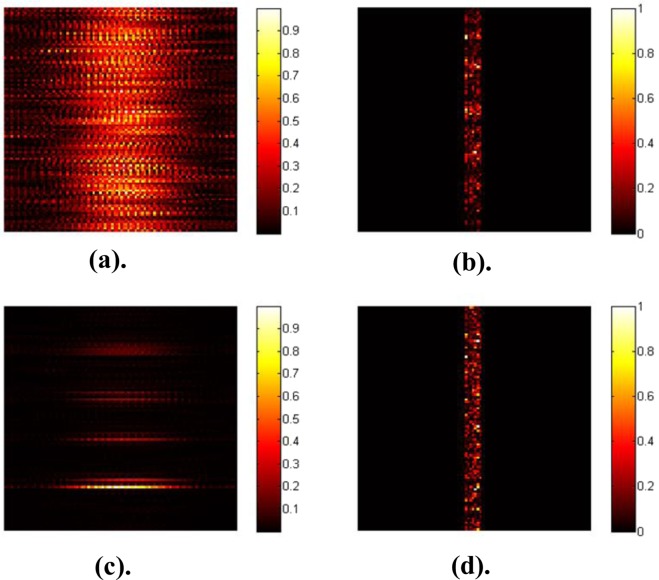
(**a**) The intensity of the field at the target plane, before optimization when all the phase variables are set to 2π (feedback from this plane is not used in the optimization). The target plane was inspected only to verify whether or not our optimization was able to converge to a focus. (**b**) Depicts the corresponding intensity of the output captured at the surface of the scatterer, after the dual pass. (**c**) Reveals the focused spot obtained, after the optimization. (**d**) Corresponding output intensity of (**c**).

In the second iteration, the *particle’s* position and velocity are updated by using the formula given below:

10 $$\begin{array}{rcl}{\rm{vel}}({\rm{k}}+1) & = & {\rm{w}}\ast ({\rm{v}}({\rm{k}})+{\rm{C}}1\ast \mathrm{rand}(0,1)\ast ({\rm{pbest}}-{\rm{particle}})\\  &  & +\,{\rm{C}}2\ast {\rm{rand}}(0,1)\ast ({\rm{gbest}}-{\rm{particle}}))\end{array}$$

11 $${\rm{particle}}({\rm{k}}+1)={\rm{particle}}({\rm{k}})+\mathrm{vel}(k+1)$$

where *w* is the inertial weight, C1 and C2 = 2 (constriction coefficient), *k* refers to the current iteration number. Now, the procedure is repeated for *k* = *MaxIt*. In our case, *MaxIt* = 4000. Figure 5(a–d) show the results of the simulation.

**Figure 6:**
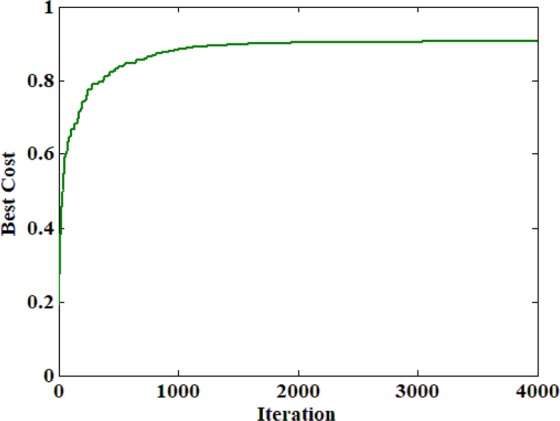
Best cost value plotted against the number of iterations.

Figure 6 displays the cost function value plotted against the number of iterations. Comparing to Fig. 5(a,c) displays the scattering has reduced, and the light has converged to a focus in 1-D. Please note: Since the optimization process implementation in 1-D was attempted, the output field captured along the horizontal direction was averaged. Hence, a focus was obtained, which is converged only along the vertical direction, and it but can still be scattered in the other direction as seen in Fig. 5(c).

**Figure 7:**
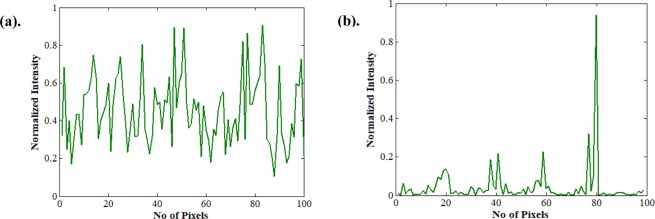
Displays intensity plot along the vertical axis of the target plane. (**a**) Before optimization. (**b**) After 1^st^ optimization.

Figure 7(a,b) display the corresponding intensity plots of Fig. 5(a,b), along the vertical direction. A sharp peak is illustrated in Fig. 7(b) indicating a converged focal spot. We can obtain another focal spot at a different spatial location by running a different set of iteration.

**Figure 8:**
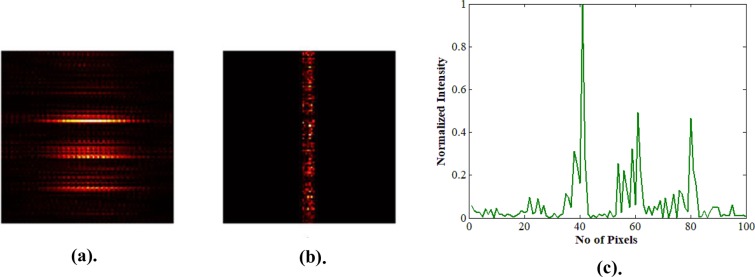
Displays the result of the second optimization. (**a**) Focus spot after 2^nd^ optimization. (**b**) Corresponding output intensity. (**c**) Corresponding intensity profile of (a).

The results of the 2^nd^ iteration are illustrated in Fig. 8(a–c). Figure 8(a) Illustrates a focal spot at spatial location being different than the previous one. In Fig. 8(c), there are two other smaller peaks observed, apart from the desired focal spot. This happens since there are more than one solution for the optimization that is being performed. Also, another point to note is the nature of the optimization scheme that we follow. We use only phase to encode the wavefront. However, if both the amplitude and phase were controlled, a much sharper peak would be obtained with good signal to noise ratio. In the supplementary material we show, a sharp focus can be obtained through simulations, when the solution space is restricted (Section S3). Mapping all such focal spots by different iteration and producing a raster scan per pixel to reconstruct the object that is behind the scattering media is possible. However, we have no control over the location of the focal spot because each focal spot location is random in each cycle of iteration. Our prime objective is to retrieve the object hidden behind the medium. It could be possible only if we map all the areas where the object reflects the light back through the medium. Hence our optimization scheme is aimed at retrieving all such points where light can focus and use this to reconstruct the object. The location of the focal spot in each iteration is random. Nevertheless, it is not considered a drawback as long as our aim is to reconstruct the object non-invasively.

As previously mentioned, the most appropriate way to show it experimentally is to perform a phase modulation. However, considering the ease with experiment we chose to perform amplitude modulation for our experimental proof of concept.

### Experimental validation

**Figure 9:**
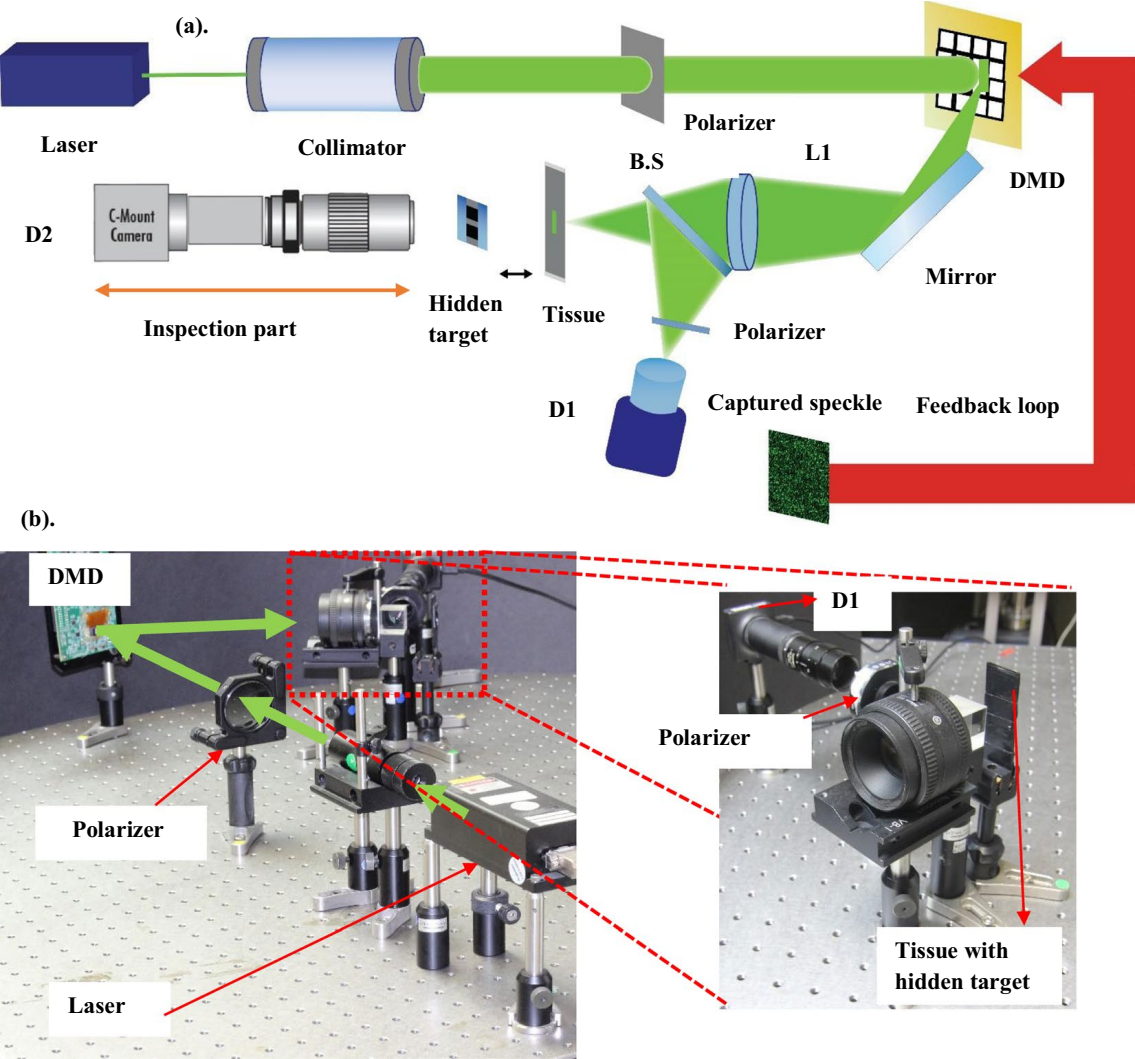
(**a**) Schematic of the experimental setup of the proposed technique in amplitude modulation scheme with the inspection part. (**b**) Picture of the real experimental setup. Inspection part is not shown in (**b**).

An amplitude modulation scheme experiment was performed. A Digital Micromirror Device (0.45 WXGA-DMD) (1280 × 800 pixels with pixel size 7.6 µm) from *Texas Instruments* was used as an amplitude modulator in the experiment. A diode pumped solid-state laser at 532 nm, collimated using a collimator passes through a polarizer and illuminates the DMD (*see* Fig. 9(a,b)).

We have used a very thin slice of chicken breast tissue of approximate thickness 20 microns as the scattering sample. It has scattering properties alike human tissue. We quantified the medium by using speckle contrast. (see the Supplementary material Fig. SM4) Ideally, our modeling should work for any thickness of the sample, provided that a sufficient signal is available to detect which comes after the dual pass through the medium. The focus spot size is dependent on the feature size of the medium. For higher resolution, one must modulate the light by de-magnifying the input light on the wavefront shaper to match the features on the medium. Hence, we can control each scattering channel more precisely, producing a focus spot with higher resolution that is still diffraction limited. On the DMD, we introduce an amplitude mask of 100 × 400 pixels to produce a line of light with amplitude modulation along the vertical direction. The amplitude values changing from 0 to 255. Along the 100 pixels, we keep the amplitude value to match. Along 400 pixels, we have amplitude modulation. We club 10 pixels together to form 40 pixels. Hence, our optimization is performed for 40 variables and the length of the line produced on the DMD is at 3 mm. Using a lens L1, an Amplitude modulated line of light is then imaged on the tissue, that passes through a beam splitter (BS), with a demagnification of 0.5. Hence, the length of the line illuminating the sample is 1.5 mm with the size of each pixel equal to 38 microns. Only the line that passes through the masked sample is allowed to pass through. The target is placed at 0.5 mm from the sample. The target in this experiment is a barcode consisting of 3 white and 3 black lines. The width of the 1^st^ and 3^rd^ white bar is 180 microns, and the second white bar is 84 microns. This white bar allows the light to passthrough it. The black bar absorbs light.

In order to get the scattered light back through the same medium, we introduced a partially transmitting mirror behind the target. This is done for inspecting the focus spot formed, after optimization. Of course, this information is not used in the optimization feedback, and it is only used for our reference to know that the demonstrated procedure works well.

Detector D1 captured the output light coming out of the medium, after dual pass. Polarizer P2 blocks mostly all the back reflection from the first surface of the sample. Also, the angle of illumination is such that we have less contribution from the specular light arising from the first surface of incidence. Detector D2 is used to inspect the focal spot produced, after the optimization. Infinity corrected microscope objective lens (Mitutoyo M Plan Apo Objective of magnification 5X and N.A = 0.14, f = 200 mm) is used to image the target plane onto the detector with the help of a tube lens.

## Results and Discussion

PSO runs for *MaxIt* = 100. Number of particles used is 20. Number of variables optimized is 40. The captured intensity of the output is captured using D1 and it has 216 × 52 pixels. It is resized to 40 × 52 pixels and averaged along the 52 pixels to get a 1-D form of the output light. It is, then, correlated against the 1-D version of intensity of the input amplitude mask applied on the DMD. This is the cost function in this optimization. The optimization produces a converged spot, after *N* = 100 iterations. This was continued for 3 cycles, until 3 converged spots were obtained on 3 white bars that are hidden behind the sample.

**Figure 10:**
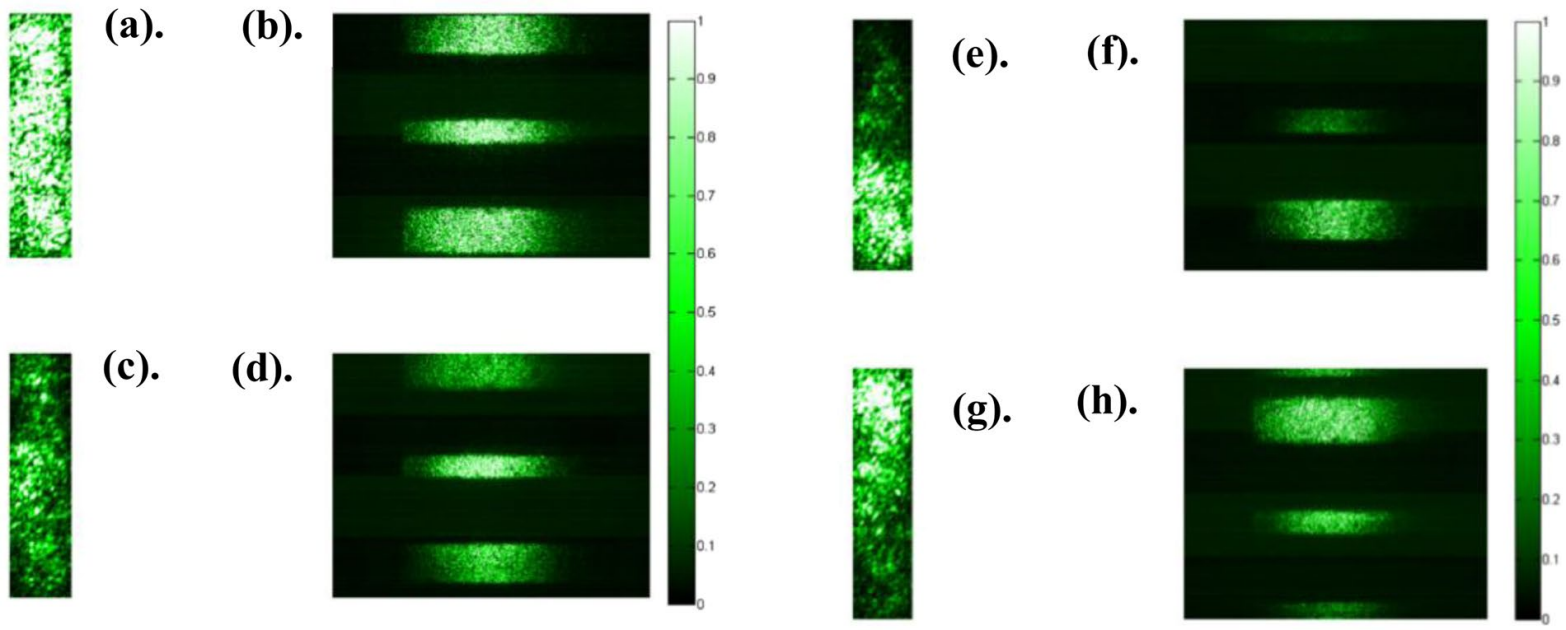
(**a**) Output light intensity captured by D1, when all the pixels on the DMD are set to 255. (**b**) Corresponding intensity on the target plane. (**c**,**e**,**g**) Output intensity captured by D1 after 1^st^, 2^nd^, and 3^rd^ cycle of optimization respectively. (**d**,**f**,**h**) Corresponding intensity on the target plane of (**c**,**e**,**g**) captured by D2.

Figure 10(a–h) illustrates the results of the optimization. Figure 10(a) displays the intensity of the output light captured when all the pixels on the DMD are set to 255. Figure 10(b) displays the corresponding intensity of the scattered light on the target plane. Figure 10(c) Reveals the intensity of the output light captured, after the first cycle of optimization. At this stage, the intensity of the input and output light is highly correlated. According to our modelling, we should have obtained a converged spot in 1-D at the target plane. The captured image from D2 is seen in Fig. 10(d), and it displays the scattering has been reduced and converged to a spot illuminating in the middle white bar. The size of the converged spot in 1-D is about 180 microns. The cycle is repeated 2 more times, and the location of the converged spot changes, as depicted in the Fig. 10(f,h). Figure 10(e,g) display the corresponding output intensity that is being captured. In order to reconstruct the 1-D target, all the 3 output intensities corresponding to the 3 converged spots that were obtained are all added.

**Figure 11:**
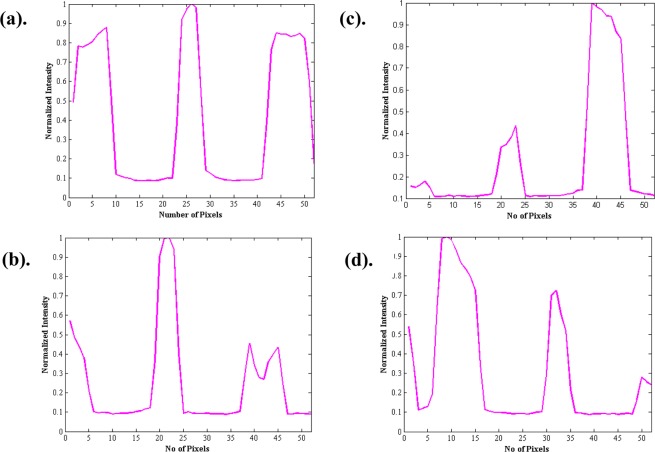
(**a**) Intensity profile on the target plane, before optimization. (**b–d**) Corresponding intensity profile on the target plane after 1^st^, 2^nd^, and 3^rd^ cycle of optimization.

Figure 11(b–d) diplay a sharp peak at the desired locations. Please note**:** This only verifies that convergence was achieved at the desired locations. However, the nature of the optimization issue of having more than one solution remains, as there are minor peaks associated with each sharp peak.

**Figure 12:**
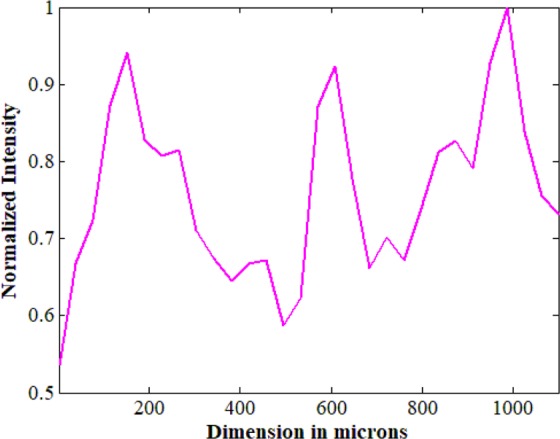
Reconstructed 1-D intensity profile of the target.

We have added all 3 ouput intensities, resize, and average along the 52 pixels to get a 1-D plot, as shown in the Fig. 12. The dimensions in microns are indicated along the x-axis, and the normalized intensity along the y-axis. The reconstructed profile indicates 3 peaks corresponding to the 3 peaks in the target lines. Full width half maximum (FWHM) of the 1^st^ peak is about 189 microns –being close to its actual dimension at 180 microns. FWHM of the 2^nd^ peak is 94 microns –being close to its original size at 84 microns. Finally, the 3^rd^ peak has a FWHM of 213 microns. All reconstructed dimensions are very close to the actual width values with small deviations.

## Conclusions

We presented a unique, reference-free, non-invasive, reverse response-based wavefront shaping technique to image through s scattering medium. Since our illumination system and the detection system are on the same side of the object, it does not require the direct access behind the medium nor use a feedback from there.

The application of our novel concept can be utilized mainly in biological imaging as a non-invasive diagnostic tool.

## Supplementary information

Supplymentary Materials

## References

[1] Goodman JW, Huntley WH, Jackson DW, Lehmann M (1966). Wavefront‐Reconstruction Imaging Through Random Media. Applied Physics Letters.

[2] Leith EN, Upatnieks J (1966). Holographic Imagery Through Diffusing Media. Journal of the Optical Society of America.

[3] Kogelnik H, Pennington KS (1968). Holographic Imaging Through a Random Medium. Journal of the Optical Society of America.

[4] Huang D (1991). Optical coherence tomography. Science.

[5] Denk W, Strickler JH, Webb WW (1990). Two-photon laser scanning fluorescence microscopy. Science.

[6] Helmchen F, Denk W (2005). Deep tissue two-photon microscopy. Nat. Methods..

[7] Gibson AP, Hebden JC, Arridge SR (2005). Recent advances in diffuse optical imaging. Physics in Medicine and Biology.

[8] Katz O, Heidmann P, Fink M, Gigan S (2014). Non-invasive single-shot imaging through scattering layers and around corners via speckle correlations. Nature photonics.

[9] Edrei E, Scarcelli G (2016). Optical imaging through dynamic turbid media using the Fourier-domain shower-curtain effect. Optica.

[10] Wu P, Liang Z, Zhao X, Su L, Song L (2017). Lensless wide-field single-shot imaging through turbid media based on object-modulated speckles. Applied Optics.

[11] Freund I (1990). Looking through walls and around corners. Physica A: Statistical Mechanics and its Applications.

[12] Freund I, Rosenbluh M, Feng S (1988). Memory effects in propagation of optical waves through disordered media. Physical review letters.

[13] Bertolotti J (2012). Non-invasive imaging through opaque scattering layers. Nature.

[14] Fienup JR (1982). Phase retrieval algorithms: a comparison. Applied Optics.

[15] Singh AK, Pedrini G, Takeda M, Osten W (2017). Scatter-plate microscope for lensless microscopy with diffraction limited resolution. Scientific Reports.

[16] Wu L-A, Hartline BK, Horton RK, Kaicher CM (2009). Lensless “Ghost” Imaging with Thermal Light Sources (abstract). AIP Conference Proceedings.

[17] Shih Y (2016). The Physics of Turbulence-Free Ghost Imaging. Technologies.

[18] Xu Y-K (2015). Is ghost imaging intrinsically more powerful against scattering?. Optics Express.

[19] Yaqoob Z, Psaltis D, Feld MS, Yang C (2008). Optical phase conjugation for turbidity suppression in biological samples. Nature Photonics.

[20] Bianco V (2012). Clear coherent imaging in turbid microfluidics by multiple holographic acquisitions. Optics Letters.

[21] Bianco V (2014). Imaging adherent cells in the microfluidic channel hidden by flowing RBCs as occluding objects by a holographic method. Lab on a Chip.

[22] Paturzo M (2012). Microscopy imaging and quantitative phase contrast mapping in turbid microfluidic channels by digital holography. Lab on a Chip.

[23] Vellekoop IM, Mosk AP (2007). Focusing coherent light through opaque strongly scattering media. Optics Letters.

[24] Popoff SM (2010). Measuring the Transmission Matrix in Optics: An Approach to the Study and Control of Light Propagation in Disordered Media. Physical Review Letters.

[25] Choi Y, Yoon C, Kim M, Choi W, Choi W (2014). Optical Imaging With the Use of a Scattering Lens. IEEE Journal of Selected Topics in Quantum Electronics.

[26] Mcdowell EJ (2010). Turbidity suppression from the ballistic to the diffusive regime in biological tissues using optical phase conjugation. Journal of Biomedical Optics.

[27] Aulbach J, Gjonaj B, Johnson PM, Mosk AP, Lagendijk A (2011). Control of Light Transmission through Opaque Scattering Media in Space and Time. Physical Review Letters.

[28] Mosk AP, Lagendijk A, Lerosey G, Fink M (2012). Controlling waves in space and time for imaging and focusing in complex media. Nature Photonics.

[29] Park J, Park J-H, Yu H, Park Y (2015). Focusing through turbid media by polarization modulation. Optics Letters.

[30] Ghielmetti G, Aegerter CM (2012). Scattered light fluorescence microscopy in three dimensions. Opt. Express.

[31] Ghielmetti G, Aegerter CM (2014). Direct imaging of fluorescent structures behind turbid layers. Opt Express.

[32] Yang X, Hsieh C-L, Pu Y, Psaltis D (2012). Three-dimensional scanning microscopy through thin turbid media. Optics Express.

[33] Katz O, Small E, Silberberg Y (2012). Looking around corners and through thin turbid layers in real time with scattered incoherent light. Nature Photonics.

[34] Katz O, Small E, Guan Y, Silberberg Y (2014). Noninvasive nonlinear focusing and imaging through strongly scattering turbid layers. Optica.

[35] Zhou EH, Ruan H, Yang C, Judkewitz B (2014). Focusing on moving targets through scattering samples. Optica.

[36] Popoff S, Lerosey G, Fink M, Boccara AC, Gigan S (2010). Image transmission through an opaque material. Nature Communications.

[37] Kennedy, J. & Eberhart, R. Particle swarm optimization. Proceedings of *ICNN95 - International Conference on Neural Networks*.

