# Author Correction: Non-Invasive Imaging Through Scattering Medium by Using a Reverse Response Wavefront Shaping Technique

**DOI:** 10.1038/s41598-020-62442-9

**Published:** 2020-04-02

**Authors:** Abhijit Sanjeev, Yuval Kapellner, Nadav Shabairou, Eran Gur, Moshe Sinvani, Zeev Zalevsky

**Affiliations:** 1 Faculty of Engineering and the Institute for Nanotechnology and Advanced Materials, Bar-Ilan University, Ramat-Gan, 5290002 Israel; 2 EKB Technologies Ltd., Bat Yam, 5951301 Israel; 3 Azrieli College of Engineering, Jerusalem, 9103501 Israel

Correction to: *Scientific Reports* 10.1038/s41598-019-48788-9, published online 22 August 2019

The Acknowledgements section in this Article was omitted. The Acknowledgements section should read:

“We acknowledge the EU’s Horizon 2020 research and innovation programme under the Marie Skłodowska-Curie grant agreement No 674901 (‘switchBoard) for funding this project.”

