# Supplementary material for: Non-Invasive Imaging Through Scattering Medium by Using a Reverse Response Wavefront Shaping Technique
Source: Sci Rep. 2019 Aug 22;9:12275. doi: 10.1038/s41598-019-48788-9 (PMC6706411; doi:10.1038/s41598-019-48788-9)
Supplement: Supplementary file 1 — Supplymentary Materials [file 41598_2019_48788_MOESM1_ESM.pdf]

Non-Invasive Imaging Through Scattering Medium by Using a Reverse Response Wavefront Shaping Technique

Abhijit Sanjeev[1,2*], Yuval Kapellner[2], Nadav Shabairou[1], Eran Gur[3], Moshe Sinvani[1] and Zeev Zalevsky[1]

[1]Faculty of Engineering and the Institute for Nanotechnology and Advanced Materials, Bar-Ilan University, Ramat-Gan 5290002, Israel
[2]EKB Technologies Ltd, Bat-Yam 59513, Israel
[3] Azrieli College of Engineering, Jerusalem 9103501, Israel
**Correspondence and requests for materials should be addressed to A.S.*
(abhijitsanjeevk@ gmail.com)

Supplementary Material

S1. Proof for Matrix A being a Unitary Matrix.

Please refer to Matrix A in Eqn. 1 in main manuscript. The matrix A is composed from product of a DFT matrix which is unitary and of diagonal matrix which is also unitary. Below is a proof that a product of unitary matrix remains unitary. Unitary matrix fulfills:

$$R^{*}R = I \tag{1}$$

Where * is complex conjugate transpose. Let us take another matrix Q which also Unitary, for which we can write:

$$Q^{*}Q = I \tag{2}$$

Then their product fulfills:

$$(QR)^{*}(QR) = R^{*}(Q^{*}Q)R = R^{*}R = I \tag{3}$$

Hence the product of two unitary matrices remain unitary.

S2. Generalization to 2D scattering:

For two-dimensional images we could write the 2D vector as longer 1D vector:

$$\begin{bmatrix} a_{1,1} & \cdots & a_{1,N} \\ a_{2,1} & \cdots & a_{2,N} \\ \vdots & \ddots & \vdots \\ a_{N,1} & \cdots & a_{N,N} \end{bmatrix} \Rightarrow \begin{bmatrix} a_{1,1} \\ \vdots \\ a_{1,N} \\ a_{2,1} \\ \vdots \\ a_{2,N} \\ \vdots \\ a_{N,1} \\ \vdots \\ a_{N,N} \end{bmatrix} \qquad (4)$$

Since 2D Fourier transform is a **separable kernel**, this the 2D Fourier transform when applied on such an 1D elongated vector could be written as follows:

$$\begin{bmatrix} a_{1,1} \\ \vdots \\ a_{1,N} \\ a_{2,1} \\ \vdots \\ a_{2,N} \\ \vdots \\ a_{N,1} \\ \vdots \\ a_{N,N} \end{bmatrix} \times \begin{bmatrix} [DFT] & \begin{bmatrix} 0 & \cdots & 0 \\ \vdots & \ddots & 0 \\ 0 & \cdots & 0 \end{bmatrix} \cdots & \begin{bmatrix} 0 & \cdots & 0 \\ \vdots & \ddots & 0 \\ 0 & \cdots & 0 \end{bmatrix} \\ \vdots & \ddots & \vdots \\ \begin{bmatrix} 0 & 0 & 0 \\ 0 & 0 & 0 \\ 0 & 0 & 0 \end{bmatrix} & \begin{bmatrix} 0 & \cdots & 0 \\ \vdots & \ddots & 0 \\ 0 & \cdots & 0 \end{bmatrix} \cdots & [DFT] \end{bmatrix} \times \begin{bmatrix} [DFT^t] & \begin{bmatrix} 0 & \cdots & 0 \\ \vdots & \ddots & 0 \\ 0 & \cdots & 0 \end{bmatrix} \cdots & \begin{bmatrix} 0 & 0 & 0 \\ 0 & 0 & 0 \\ 0 & 0 & 0 \end{bmatrix} \\ \vdots & \ddots & \vdots \\ \begin{bmatrix} 0 & 0 & 0 \\ 0 & 0 & 0 \\ 0 & 0 & 0 \end{bmatrix} & \begin{bmatrix} 0 & \cdots & 0 \\ \vdots & \ddots & 0 \\ 0 & \cdots & 0 \end{bmatrix} \cdots & [DFT^t] \end{bmatrix} \qquad (5)$$

Where $[DFT]$ is the DFT matrix performing 1D Fourier transform (designated as $[F_{ij}]$ in Eq. 1 in main manuscript).

The free space propagation matrix appearing in Eq. 1 in the main manuscript (the one that multiplied the spectrum of the object) as well as the phase matrix are both diagonal matrixes. They will remain diagonal matrixes for the formulation of the longer 1D vector containing the formulation of the 2D object. Thus, the assumption of using unitary property in order to extract the algorithm convergence is also true for the 2D case. In section S3, apart from the 1D simulation results, we also show simulation for 2D case.

S3. Additional Simulation Results

In the main manuscript, we show that we perform the optimization for a predefined number of iterations (MaxIt). However, here we show that it is also possible to perform iteration based on a predefined cost function value below which the iteration continues and above which the iteration terminates. Please refer Fig. SM1 for the flowchart of the steps involved in the optimization. Cost function in our case is the phase correlation between $E_{in_j}(x_j)$ and $E_{out_j}{}^*(x_j)$. We set the max correlation to be 0.85. This will serve as the decision criteria for the iterations.

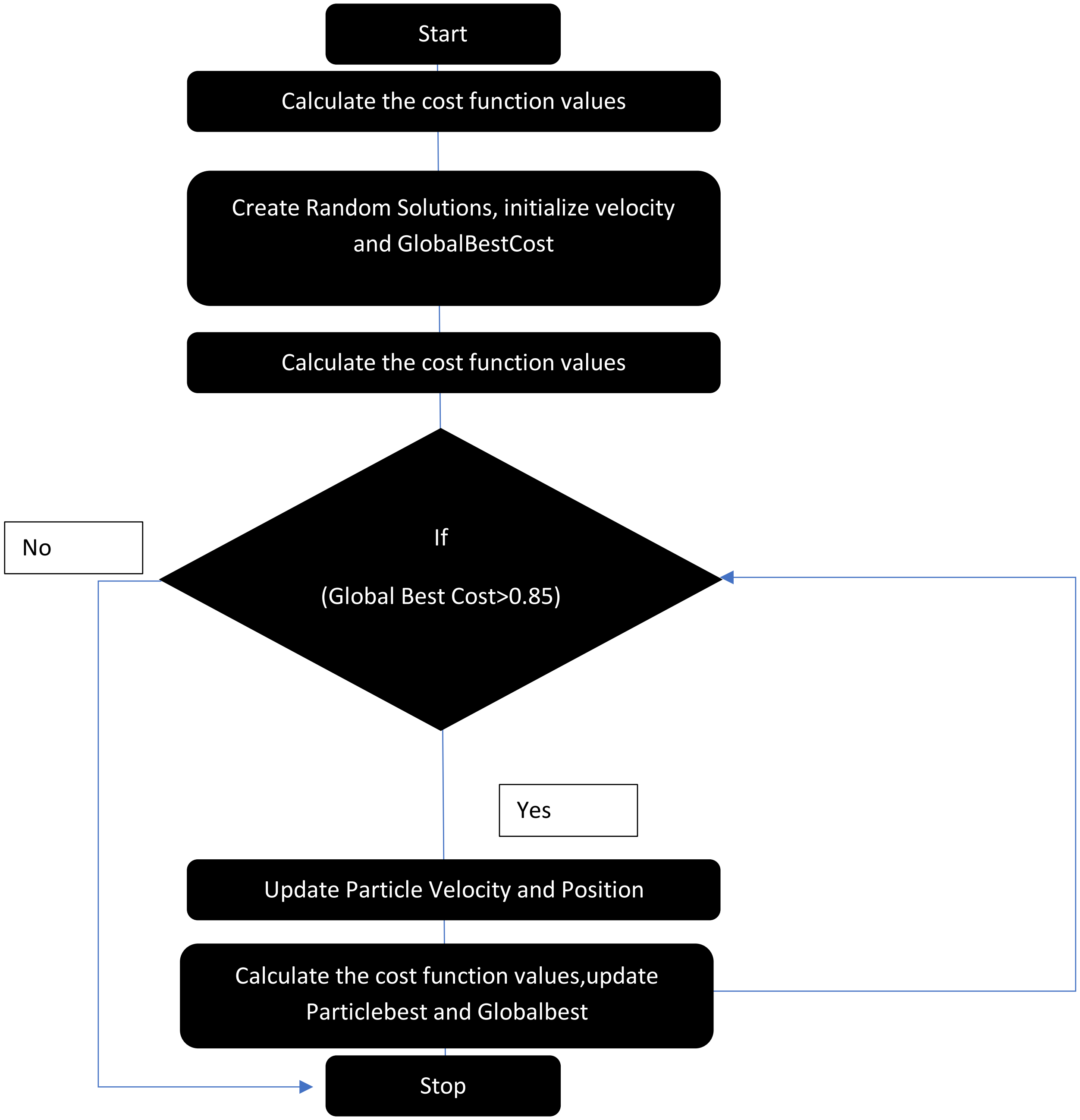

Figure SM1 : Flowchart of the Optimization

We have kept the same simulation parameters as in the main manuscript. Using this simulation, we would also like to show that when the solution space is restricted, we get a sharp focus with much lower peaks at other locations. To perform this, we use two black and white lines each of 10 pixels width in the target plane whereas we used 5 such white spaces in the main manuscript. In another set of iteration, we restricted the target plane by one white space.

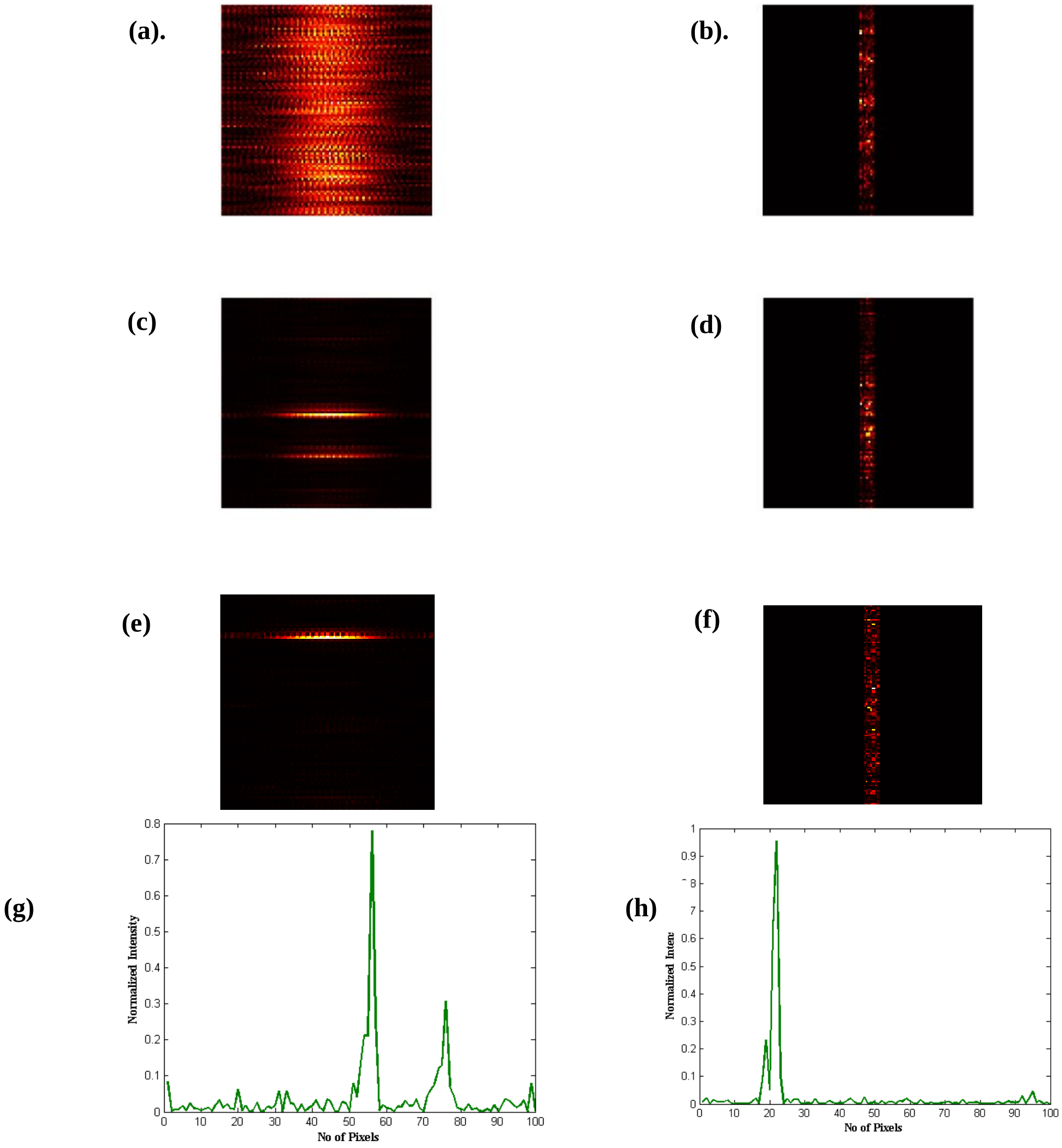

Figure SM2 : (a). The intensity of the field at the target plane before optimization when all the phase variables are set to 2π (feedback from this plane is not used in the optimization). (b). Shows the corresponding intensity of the output captured at the surface of the scatterer after the dual pass. (c). Shows the focused spot obtained after the optimization when we have two white spaces at target plane. (d). Corresponding output intensity of (c). (e). Shows the focused spot obtained after the optimization when we have one white spaces at target plane. (f). Corresponding output intensity of (e). (g) Intensity profile plot of (c). (h) Intensity profile plot of (e).

The results of the optimization are shown in Fig. SM2. It is clearly seen from Fig. SM2 (g) and (h) that when solution space (target plane) is restricted we get a sharp focus with lower sub peaks occurring in other locations.

Simulation Results for 2D Scattering

We considered 10 X 10 pixels for simulation. Phase Scattering matrix has a circular gaussian distribution of random complex numbers. Short Fresnel propagation was used for propagation of light from the scatterer to the target plane and back from target plane to the scatterer. Target plane is placed at 1 mm from the scatterer. The 10X10 pixels is zero padded such that total pixels are 50x50. As already discussed, each 2D matrix can be considered as a long 1D matrix. Hence, our input E_{in}, which is a 10X10 pixels matrix, can be considered as a 100 pixels long 1D vector. It means number of variables for optimization equals 100. Output field after the dual pass (E_{out}) was treated in the same manner as 1D long vector. Optimization is done to increase the phase correlation between the E_{in} and E^*_{out}. The decision criterion for optimization (Global Best Cost) is fixed at 0.9. We get a sharp focus at the target plane after optimization. (Figure SM3)

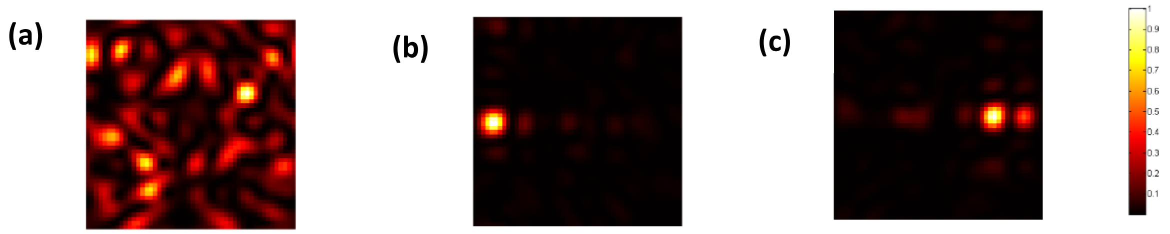

Figure SM3: (a) Target Plane Intensity before optimization. (b) Focused spot at the target plane after 1st cycle of optimization. (c) Focused spot at the target plane after 2nd cycle of optimization.

S4. Scattering Tissue Properties

In our experiment, we have used a thin slice of chicken breast tissue (approximately 20 microns thickness) as the scattering sample. We performed the speckle contrast analysis of the speckle formed after dual pass through the tissue (Figure SM4).

Speckle contrast is given by :

$$S = \frac{\sigma}{< \mathrm{I} >}$$

Where, σ is the standard deviation of the speckle intensity and $< I >$ the ensemble average of the intensity, here the spatial average of the intensity. Our tissue is acting as a weakly scattering medium since the speckle contrast is 0.37.

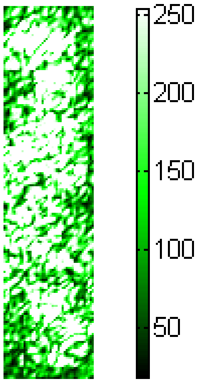

Figure SM4 : The Speckle intensity captured after dual pass through the medium.
